# Analysis of the Water Demand-Supply Gap and Scarcity Index in Lower Amu Darya River Basin, Central Asia

**DOI:** 10.3390/ijerph19020743

**Published:** 2022-01-10

**Authors:** Zheng Wang, Yue Huang, Tie Liu, Chanjuan Zan, Yunan Ling, Chenyu Guo

**Affiliations:** 1 State Key Laboratory of Desert and Oasis Ecology, Xinjiang Institute of Ecology and Geography, Chinese Academy of Sciences, Urumqi 830011, China; wangzheng19@mails.ucas.ac.cn (Z.W.); liutie@ms.xjb.ac.cn (T.L.); zanchanjuan18@mails.ucas.ac.cn (C.Z.); lingyunan18@mails.ucas.ac.cn (Y.L.); guochenyu16@mails.ucas.ac.cn (C.G.); 2 State Key Laboratory of Remote Sensing and Geographic Information System Application, Urumqi 830011, China; 3 University of Chinese Academy of Sciences, Beijing 100049, China; 4 Research Center for Ecology and Environment of Central Asia, Chinese Academy of Sciences, Urumqi 830011, China; 5 China-Pakistan Joint Research Center on Earth Sciences, CAS-HEC, Islamabad 45320, Pakistan

**Keywords:** lower reaches of the Amu Darya River Basin, crop water requirement, ecological water demand, water demand-supply gap, water scarcity index

## Abstract

Lower reaches of the Amu Darya River Basin (LADB) is one of the typical regions which is facing the problem of water shortage in Central Asia. During the past decades, water resources demand far exceeds that supplied by the mainstream of the Amu Darya River, and has resulted in a continuous decrease in the amount of water flowing into the Aral Sea. Clarifying the dynamic relationship between the water supply and demand is important for the optimal allocation and sustainable management of regional water resources. In this study, the relationship and its variations between the water supply and demand in the LADB from the 1970s to 2010s were analyzed by detailed calculation of multi-users water demand and multi-sources water supply, and the water scarcity indices were used for evaluating the status of water resources utilization. The results indicated that (1) during the past 50 years, the average total water supply (TWS) was 271.88 × 10^8^ m^3^/y, and the average total water demand (TWD) was 467.85 × 10^8^ m^3^/y; both the volume of water supply and demand was decreased in the LADB, with rates of −1.87 × 10^8^ m^3^/y and −15.59 × 10^8^ m^3^/y. (2) percentages of the rainfall in TWS were increased due to the decrease of inflow from the Amu Darya River; percentage of agriculture water demand was increased obviously, from 11.04% in the 1970s to 44.34% in 2010s, and the water demand from ecological sector reduced because of the Aral Sea shrinking. (3) the supply and demand of water resources of the LADB were generally in an unbalanced state, and water demand exceeded water supply except in the 2010s; the water scarcity index decreased from 2.69 to 0.94, indicating the status changed from awful to serious water scarcity. A vulnerable balanced state has been reached in the region, and that water shortages remain serious in the future, which requires special attention to the decision-makers of the authority.

## 1. Introduction

In arid and semi-arid areas, water resources restrict the development of the regional ecosystems and social-economic [1]. Reasonable allocation of limited water resources and improvement of utilization efficiency is the key to alleviating water resources shortage and promoting social-economic development [2]. Under the dual impact of climate change and human activity, the water supply from the Amu Darya River was continuously decreased, while the water demand sharply increased with the development of the national economy, particularly for the increase of irrigation water. The relationship between the water supply and demand was changed in different historical periods. Clarifying the dynamic relationship between the water supply and water demand is an important basis for the sustainable management of water resources in arid inland river basins.

Amu Darya River, one of the two main sources of the Aral Sea, is a typical inland river in Central Asia, with a length of 2540 km, originates in the high mountains and glacier of the Pamir-Alay Plateau and the Hindu Kush Mountains [3]. Since the 1960s, a large amount of runoff from the Amu Darya River has been used for agricultural irrigation, and inflow to the Aral Sea has been sharply reduced. Under the special climatic conditions in arid areas, the ecosystem is extremely sensitive to the change of water resources [4]. Particularly in the middle and lower reaches of the Amu Darya River Basin, large-scale land expansion and extensive water resources utilization mode lead to problems such as the deterioration of the ecological environment in oasis-desert areas, and the ecological environment in the Aral Sea region continues to deteriorate [5]. It has received extensive attention from scholars around the world [6,7,8,9,10]. Veldwisch and Spoor [6] indicated that the human and financial investment for water resources management in Uzbekistan were inadequate, resulting in poor functioning of the irrigation drainage network. Trevisani [7] presented that there was a problem of unequal distribution of water resources in the lower Amu Darya River Basin (ADRB), due to water resources were treated as an asset to be allocated autonomously by water managers (governors or chairmen of water management associations, etc.), and it is necessary to create benefits for themselves or to trade to achieve national production goals from the point of view of government institutions. Bobojonov [11] suggested introducing water price into agricultural water to improve water use efficiency in Khorezm and improve decision-making flexibility at the farm level for economic and ecological development simultaneously. Ikramova et al. [5] developed an information-program synthesis based on methods of water balance, which considering water quantity and quality, but this model was relatively rough and does not distribute water to each water uses specifically. Tischbein et al. [8] analyzed the distribution of water resources in the irrigation area from two socio-political and ecological vegetation perspectives, and also from a multi-level perspective of the country, the state region, and farmers. Liu et al. [9] analyzed the quantitative relationship among the water balance elements in the oasis and their impacts on the Aral Sea located in the LADB. Khaydar et al. [10] accurately calculated the water demand of each crop and agricultural water demand according to the FAO Penman–Monteith method in the LADB, and preliminarily discussed the relationship between agricultural water consumption and regional water resources. However, previous studies have mostly proposed directions and targets for water resources regulation in the basin from a macro policy perspective, or analyzed the variation and efficiency of agricultural water; few researchers focused on the balance between water demand from multi-users (i.e., agricultural, municipal, industrial and ecological) and multi-sources of water supply (i.e., rainfall and river inflow), lack of practical solutions to optimize the water under such complex water supply and demand conditions.

Therefore, the object of this study is to (1) calculate the multi-users water demand in the LADB over the past 50 years, and analyze the changes in water resources consumption by using statistical, land use and land cover (LULC) and meteorological data; (2) investigate the trend of water supply in the study area based on the time series of observed precipitation streamflow data; (3) discuss the profit and loss interaction between water demand and water supply. This paper will provide scientific data support for local water managers to make decisions on sustainable water resources management in the LADB and the Areal Sea region.

## 2. Materials and Methods

### 2.1. Study Area

**Figure 1:**
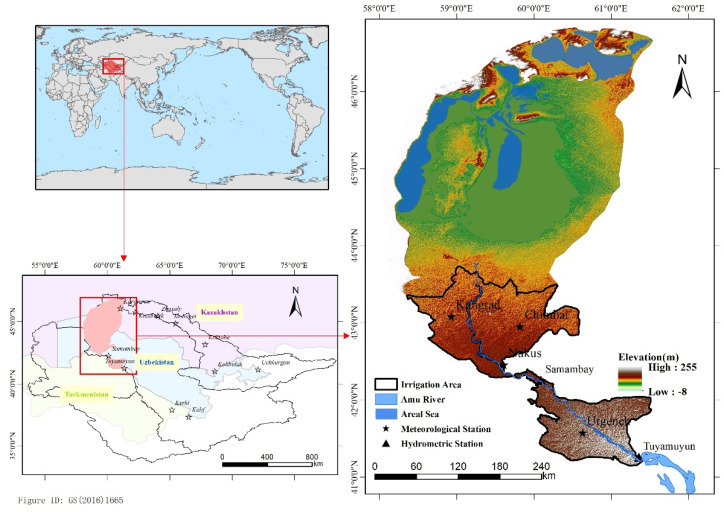
The location of the study area and its meteorological and hydrometric stations.

LADB was located within the administrative regions of Karakalpakstan and Khorazm in northern Uzbekistan, the geographical location of the study area is between 58°1′ E–61°31′ E and 41°8′ N–46°52′ N, with a total area of 96,084 km^2^. Since 21 July 2003, the previous water management system (administrative-territorial) was replaced with a basin principle of irrigation systems management. The Ministry of Agriculture and Water Resources divided the country into several large river basins, named the Basin Management of Irrigation Systems (BUIS), several Irrigation System Management Organizations (UISs) were included in each BUIS. The study area includes the lower Amu Darya River BUIS, which is one of the largest basin management areas in Uzbekistan, Aral Sea, and its surrounding ecological area. The annual average temperature was about 5–7 °C, the annual precipitation was less than 200 mm, and the average evaporation was 1100 mm–1300 mm per year. The overall topography is high in the east and low in the west, and the elevation is −8~255 m. The land-use types in the study area include cultivated, woodland, grassland, water, and wetlands, etc. Among them, cultivated land is mainly distributed in the irrigation area, grassland and woodland are widely distributed around the Aral Sea and the irrigation area, and wetlands are mainly distributed in the transition zone between the irrigation area and the Aral Sea. There are four meteorological stations and two hydrological stations in the study area. The Tuyamuyun hydrological station upstream of the lower Amu Darya River BUIS can be selected as the inlet station of the study area (Figure 1).

### 2.2. Dataset

Meteorological data from 1970 to 2019 at the Kungrad, Chimbay, Nukus, and Urgench stations were obtained for calculating the reference evapotranspiration by CROPWAT from the National Oceanic and Atmospheric Administration (NOAA), precipitation data from the Climatic Research Unit (CRU) of the University of East Anglia, CRU data have been verified by numerous studies in Central Asia [12,13] (Table 1). The crop coefficient (Kc) of each crop type was adjusted according to the guidelines for calculation of crop evapotranspiration of the Food and Agriculture Organization of the United Nations (FAO 56) and combined with the actual situation of the study area, including sowing date, harvest date, days from early, middle, and late growth stages [10]. Crop planting pattern (CPP) data were mainly obtained for calculating the agriculture water demand accurately from the statistical yearbook of the Republic of Uzbekistan and the Central Asia Water green project. Cotton, wheat, and rice were the major crops in the LADB. Since 2000, CPP varied considerably among the ten UISs. For the four UISs in the northern part, cotton and wheat were the main crop types, for the six UISs in the southern part, cotton and rice were the major crop types.

The LULC map in 1970, 1990, 2000, 2010, and 2015 was collected for obtaining the area of farmland and natural vegetation, from the “Earth System Science Data Sharing Platform—Xinjiang and Central Asia Scientific Data Sharing Platform” of the Chinese Academy of Sciences. The spatial resolution of the data is 30 × 30 m.

Runoff from the Amu Darya River is the main source of water in the study area. Tuyamuyun station at the entrance of the irrigation area was selected to measure the runoff of the Amu Darya River. Considering that part of the runoff observed by Tuyamuyun flows to Dashoguz in Turkmenistan, 50% of the runoff flows into the study area according to the water agreement between Uzbekistan and Turkmenistan [14].

### 2.3. Calculation of Agriculture and Ecology Water Demand

#### 2.3.1. Crop Water Requirement

The FAO Penman–Monteith model has been considered the universal standard to estimate reference evapotranspiration (${ET}_{0}$) and was widely used in the absence of measured data [19,20,21]. The variables used for the calculation of ${ET}_{0}$ included monthly maximum and minimum temperature, humidity, and wind speed in this study. The monthly ${ET}_{0}$ value was calculated by CROPWAT 8.0 software. The calculation formula is listed as follows:

(1) $${ET}_{0}=\frac{0.408∆\left(R_{n}-G\right)+\gamma \frac{900}{t+273}U_{2}{(e}_{s}-e_{a})}{∆+\gamma (1+0.34U_{2})}$$

(2) $$∆=\frac{4.098\left[0.6108\exp(\frac{17.27t}{t+237.3})\right]}{{\left(t+237.3\right)}^{2}}$$

where $ET_{0}$ is the reference evapotranspiration (mm); $R_{n}$ is the net radiation at the crop surface (MJ/m^2^); G is the soil heat flux density (MJ/m^2^), which is considered as zero; t is the monthly mean air temperature at 2 m height (°C); $U_{2}$ is the wind speed at 2 m height (m/s); $e_{s}$ is the saturation vapor pressure (kPa); $e_{a}$ is the actual vapor pressure (kPa); $e_{s}-e_{a}$ is the saturation vapor pressure deficit (kPa); ∆ is the slope vapor pressure curve (kPa/°C); and γ is the psychometric constant (kPa/°C), which is considered as 0.0655 kPa/°C.

Crop Water Requirement (CWR) refers to the water loss of crops due to evapotranspiration, which is deducted from the actual evapotranspiration of crops and is equal to the actual total evapotranspiration of crops in the growing season, which can be calculated by Equation (3):

(3) $$CWR=ET_{a}=K_{c}\times ET_{0}$$

where $ET_{a}$ is the crop actual evapotranspiration (mm); $K_{c}$ is the crop coefficient at a specific growth stage (Table 2).

Due to the difference in CPP among the UISs, the CWR varies by UISs [10,22], accordingly, we calculated the regional crop water requirement (*CWR_reg_*) in different UISs using Equation (4):

(4) $$CWR_{reg}=\frac{\sum ET_{a}\times A_{i}}{A}$$

where $CWR_{reg}$ is the regional crop water requirement (mm), $A_{i}$ is the planting area of crop type i (km^2^), and A is the total crop area in the UISs (km^2^). 

Effective precipitation ($P_{eff}$) is the fraction of the total precipitation as rainfall and snow melt that is available for crop use [23,24], $P_{eff}$ were calculated as follows:

(5) $$P_{eff}=\{\begin{array}{ll} \frac{P\times \left(125-0.2\times 2\times P\right)}{125} & if\;P\leq \frac{250}{3}\;\mathrm{mm}\\ \frac{125}{3}+0.1\times P & if\;P>\frac{250}{3}\;\mathrm{mm} \end{array}$$

where $P_{eff}$ is the effective precipitation (mm); P is the monthly precipitation (mm).

The net irrigation requirement (NIR) is the water amount required for the growth of the crop and was calculated by Equation (6). Then, to compare with the real irrigation volume of each UIS, we calculated the regional net irrigation requirement ($NIR_{reg}$) and irrigation water requirement ($IWR_{reg}$) by the Equations (7) and (8). The agricultural water demand (*AWD*) for the whole region is obtained by Equation (9).

(6) $$NIR=CWR-P_{eff}$$

(7) $$NIR_{reg}=CWR_{reg}-P_{eff}$$

(8) $$IWR_{reg}=NIR_{reg}\times A$$

(9) $$AWD=IWR=\sum IWR_{reg}$$

where $NIR_{reg}$ is the regional net irrigation requirement for each UIS (mm); $IWR_{reg}$ is the regional irrigation water requirement (m^3^); IWR is a summary of the $IWR_{reg}$ of UISs (m^3^); AWD is the Agricultural water demand(m^3^); Other meaning of variables as above.

#### 2.3.2. Ecological Water Demand

The quota area method was used to calculate the ecological water demand (EWD) of woodland, grassland, wetland, and water. The area of a certain type of vegetation in a certain area is multiplied by its ecological water demand quota to get the EWD of that vegetation type, and the sum of EWD of each type of vegetation in the area is the total ecological water demand of vegetation, which can be calculated by Equation (10):

(10) $$Q=\overset{n}{\underset{i=1}{\sum }}Q_{i}=\overset{n}{\underset{i=1}{\sum }}A_{i}\times R_{i}$$

where $Q_{i}$ is the vegetation ecological water demand of type i, m^3^; $A_{i}$ is the area of type i, km²; $R_{i}$ is the quota of type i, mm. The criteria for the quotas of ecological water demand of vegetation were based on the previous studies in similar inland river basins [25].

### 2.4. Balance of Water Resources Supply-Demand

Water balance explores the relationship between water availability under natural conditions and demand for water in socio-economic environments. According to the characteristics of incoming water and water consumption departments, the water balance formula is established based on the available data:

(11) $$P+R_{in}-R_{out}-\sum WD_{i}=∆GSD$$

where P is precipitation, mm; $R_{in}$ is the inflow from the ADR, which is also the main source of water in the study area, m^3^; $R_{out}$ is the outflow of water volume from the area, combined with the situation of the study area, $R_{out}$ = 0; $\sum WD_{i}$ is the amount of water required by each department, i present the water use department of agriculture, ecology, industry and municipal; ∆GSD is the residual term of water balance, and mean the gap of supply-demand, positive values represent surplus and vice versa, within a closed watershed, the ∆GSD can be considered as the volume of groundwater change.

### 2.5. Water Scarcity Index

Water scarcity is defined as a situation where insufficient water resources are available to satisfy long-term average requirements [26]. Several methods have been developed to assess water scarcity according to water quantity and water quality, for example, Falkenmark index, Criticality ratio, International Water Management Institute (IWMI) indicator, water poverty index, blue water availability, and green water availability [27,28,29,30,31,32,33,34,35]. In general terms, water scarcity represents the overexploitation of water resources when water demand is higher than water availability. Therefore, the water scarcity index (WSI), which means the ratio of water supply to water demand and has been widely used [36,37,38,39], was applied to calculate the dynamic relationship between water supply and water demand in this paper.

(12) $$WSI=\frac{W_{d}}{W_{s}}$$

where $W_{d}$ is water demand, m^3^; $W_{s}$ is water supply, m^3^. The standards for *WSI* are shown in Table 3 [40].

## 3. Results

### 3.1. Total Water Supply

**Figure 2:**
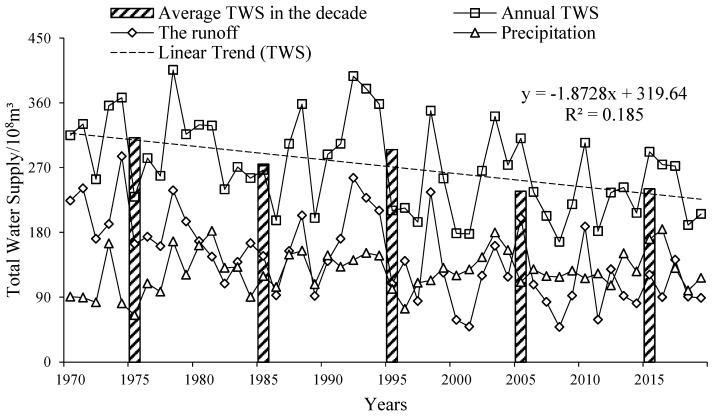
Variation of Total Water Supply during 1970 to 2019. Notes: TWS, total water supply.

The text continues here. Figure 2 shows the change of total water supply (TWS) during the 1970s to 2010s. The TWS can be divided into three parts, including the inflow from the Amu Darya River, the precipitation, and the groundwater. However, the data of groundwater are not yet available and were calculated in the balance of supply-demand as the residual item [9,18,41].

In the past 50 years, the average TWS was 271.88 × 10^8^ m^3^, in which the runoff and precipitation accounted for 53.57% and 46.43%, respectively. The change of runoff was variable with time, the average runoff was 145.66 × 10^8^ m^3^, showing a fluctuating decrease trend with a rate of 2.32 × 10^8^ m^3^/y. In the 2010s, the amount of water flowing into the irrigation area and the Aral Sea was about 40% of that in the 1970s. The annual average precipitation was 126.22 × 10^8^ m^3^, and the precipitation showed a trend of fluctuating increase with a rate of 0.51 × 10^8^ m^3^/y, in which precipitation increased sharply in the 1970s to 1980s.

### 3.2. Total Water Demand

#### 3.2.1. Agricultural Water Demand

Table 4 presents statistics on the area of different land-use types and their percentages in different periods. The percentage of the cultivated land area increased rapidly from 10.79% in 1970 to 13.7% in 1990, and then slightly decreased to 13.25% in 2010. In 2015, the area of cultivated land reached 13,908.31 km^2^, and the expansion of the cultivated was mainly on both sides of the Amu Darya River. The area of water decreased by 54,931.55 km^2^ mainly due to the shrinkage of the Aral Sea; the area of unutilized land increased by 53,243.66 km^2^, indicating that the conversion of water to unutilized land had occurred, which were the two land types with the highest degree of land change in the region, while other land types have changed to a lesser degree.

**Figure 3:**
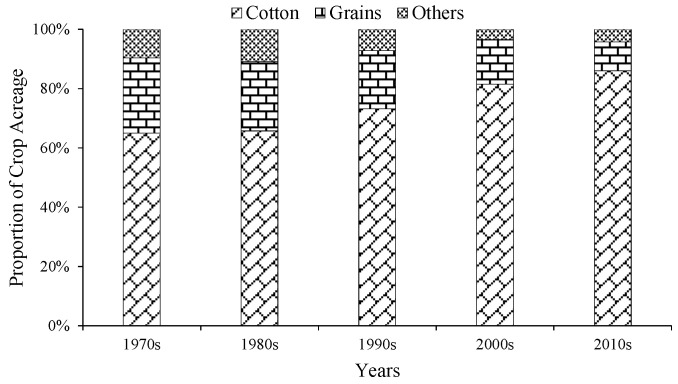
Variation of crop planting pattern during the 1970s to 2010s.

From the 1970s to 2010s, the CPP in the irrigation area has experienced significant changes, with statistics according to three major categories: cotton, grains (including wheat, rice, and maize), and others (including sunflower, vegetables, orchard, and melon) (Figure 3). The proportions of cotton sharply increased, from 65% to 86%, conversely the proportions of grain and others crop down gradually by 16% and 5%, respectively.

**Figure 4:**
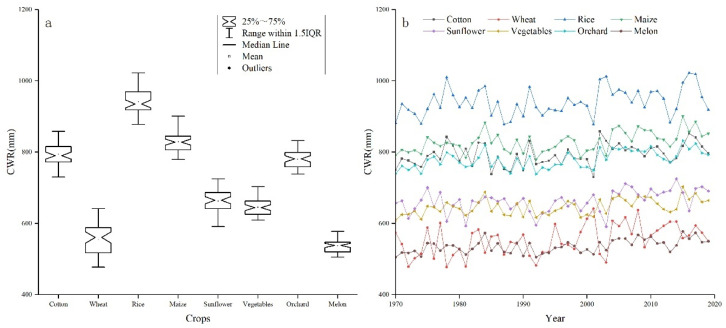
Crop water requirement for main crop types during the 1970s to the 2010s. Notes: (**a**) Distribution of CWR for crops; (**b**) The CWR temporal evolution for each crop.

CWR values during 1970–2019 were calculated separately for different crop types. As shown in Figure 4, cotton, rice, maize, and orchard crop fields had the biggest water consumption, in which the average CWR values during the growing season in the LADB were 792.89, 941.21, 829.25, and 779.87 mm, respectively. The average CWR for melon fields was 536.2 mm, which was the lowest value in the study area. The average CWR values for wheat, vegetables, and sunflower were 553.80, 646.09, and 662.37 mm, respectively.

**Figure 5:**
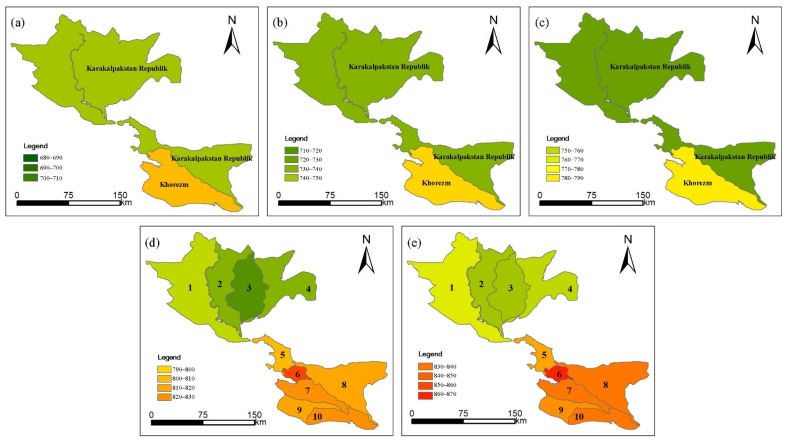
Variations of the CWRreg in the LADB in the past 50 years. (1970s (**a**);1980s (**b**); 1990s (**c**); 2000s (**d**); 2010s (**e**)). Results were displayed according to two zones before the 2000s, Karakalpakstan Republik and Khorezm; results were then displayed according to the new 10 UISs. 1 Suenli; 2 Kattagar-Bozatau; 3 Kizketken-Kegeyli; 4 Kuanishjarma; 5 Mangit-Nazarkhan; 6 Koramazi-Kilichniyozboy; 7 Shovot-Kulovot; 8 Pakhtaarna-Nayman; 9 Polvon-Gazavot; 10 Toshsoka.

The results show that before the UISs policy was implemented (Figure 5), the CWRreg of the Republic of Karakalpakstan was lower than that of Khorem. After the implementation of UISs, the CWRreg of the four UISs in the northern are lower than that of the six UISs in the southern. Kizketken-Kegeyli has the lowest CWRreg with an average of about 702.41 mm, while the Koramazi-Kilichiyozboy has the highest CWRreg with an average of 850.16 mm. 

**Figure 6:**
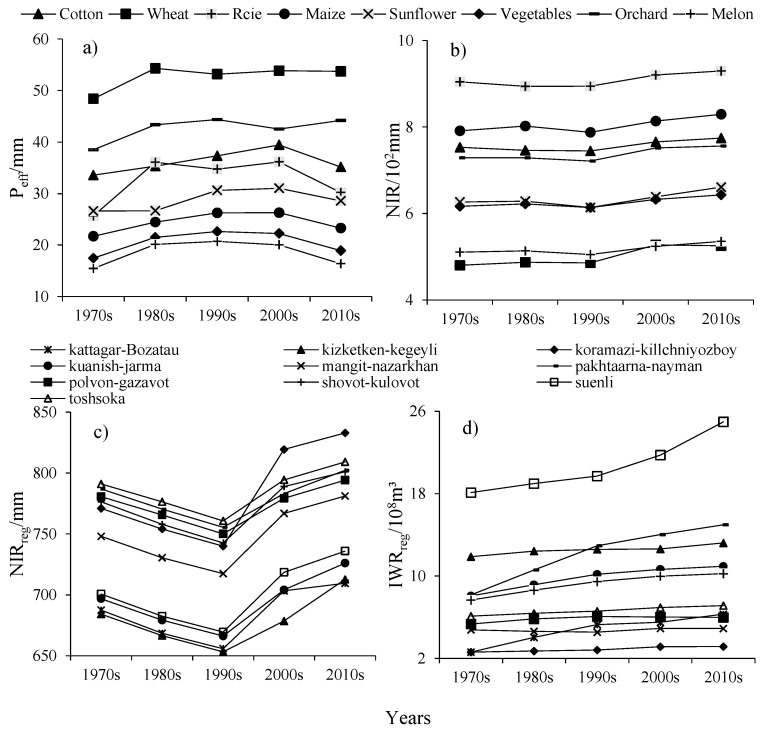
Variations of Peff (**a**) and NIR (**b**) for different crop types; NIRreg (**c**) and IWRreg (**d**) in the UISs during the 1970s to the 2010s. Notes: $P_{eff}$, the effective precipitation; NIR, net irrigation requirement; $NIR_{reg}$, regional net irrigation requirement; $IWR_{reg}$, regional irrigation water requirement.

Peff was calculated for all crops ranging from 2.41 mm to 185.91 mm; the average Peff of wheat was the highest, which was 106.94 mm; and the average Peff of melon and vegetable was the lowest, which were 19.18 mm and 22.16 mm, respectively (Figure 6a). Combined with CWR and Peff, it can be known that the NIR of wheat and melon was the lowest, which are 501.37 mm and 517.67 mm, respectively, while the NIR was highest for rice and corn, which were 908.64 mm and 804.89 mm, respectively (Figure 6b). Combined with the CPP of each UISs, the NIRreg (Figure 6c) and IWRreg (Figure 6d) were calculated. The UIS named Suenli has the highest average IWRreg of 20.70 × 10^8^ m^3^. The Koramazi-Killchniyozboy had the lowest average IWRreg of 2.88 × 10^8^ m^3^. From Table 4 and Figure 6d, with the increase of cultivated land area, the IWRreg also shows an increasing trend, and there is a good consistency between cultivated land area and IWR.

**Figure 7:**
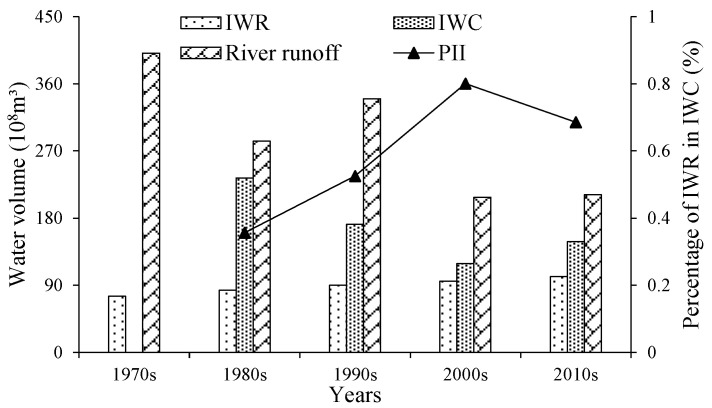
Comparison of the irrigation water consumption and inflow from the ARD during 1970–2019. Notes: IWR, irrigation water requirement; IWC, irrigation water consumption; PII, percentages of IWR in IWC.

Due to the use of field flooding irrigation in irrigated areas, irrigation water consumption (IWC) and water resource are huge and greatly wasted. This paper only calculated the percentages of IWR in IWC (PII) after the 1980s, since the data of IWC in irrigated areas before 1986 were not obtained, as shown in Figure 7, the PII was the lowest in the 1980s and the highest in the 2000s, which was only 35.7% in the 1980s, increased to 52.5% in the 1990s, reached 80.1% in the 2000s, and reached 68.6% in the 2010s. PII shows a trend of increase, which is the dual impact of increasing IWR and decreasing IWC. The reason why the PII was high in the 2000s was that the surface runoff decreased sharply from the 1990s to the 2000s, leading to a sharp decrease in IWC.

#### 3.2.2. Ecological Water Demand

**Figure 8:**
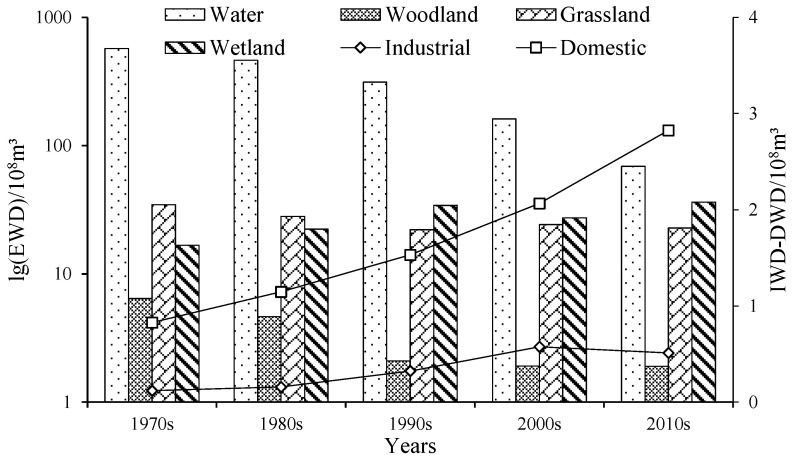
Variations of the EWD, DWD, and IWD on average in the study area during the 1970s to the 2010s. Notes: EWD, ecological water demand, including water, woodlands, grasslands, and wetlands; IWD, industrial water demand; DWD, domestic water demand.

Over the past 50 years, the ecological water demand of water, woodland, and grassland have all shown a decreasing trend except for wetland, but there were some differences (Figure 8). The EWD was 762.53 × 10^8^ m^3^ during the 1970s; due to the Aral Sea shrinking and the area of water decreasing sharply, the EWD gradually reduced to 98.92 × 10^8^ m^3^ in 2019 years, the average EWD over the past 50 years was 372.66 × 10^8^ m^3^. In the past 50 years, the EWD has decreased with a rate of 13.06 × 10^8^ m^3^/y; the average EWD of grassland and woodland were 25.54 × 10^8^ m^3^ and 3.19 × 10^8^ m^3^, respectively, and has shown a fluctuating trend of decrease, with a decrease rate of 0.27 × 10^8^ m^3^/y and 0.12 × 10^8^ m^3^/y, respectively; the average EWD of wetland was 12.45 × 10^8^ m^3^, increased dramatically in the 1970s to 1990s with an increased rate of 0.88 × 10^8^ m^3^/y, and then showed an increasing trend of fluctuation.

#### 3.2.3. Domestic and Industrial Water Demand

During the 1970s to the 2010s, both domestic water demand (DWD) and industrial water demand (IWD) showed an increasing trend (Figure 8). Where DWD increased sharply, with an increase of 2.12 × 10^8^ m^3^ and a rate of 0.42 × 10^8^ m^3^/y, the reasons for the rapid increase were positively correlated with population growth and urbanization. The average IWD was not high during the last 50 years compared to DWD, increased slightly from the 1970s to 1980s, increased significantly from the 1980s to 2000s, and then remained stable. The average IWD in the past 50 years was 0.34 × 10^8^ m^3^, which was about 1/5 of the average DWD. In combination with the agriculture-oriented industry characteristics of the Republic of Uzbekistan, the industrial economy has a small share, so the IWD was low. In addition, the Growth Domestic Product (GDP) changes in the past 50 years show that the GDP was always low before the 1990s, but after the 1990s, the GDP began to grow continuously, so the IWD also increases with the change of GDP.

#### 3.2.4. Change of the Total Water Demand

**Figure 9:**
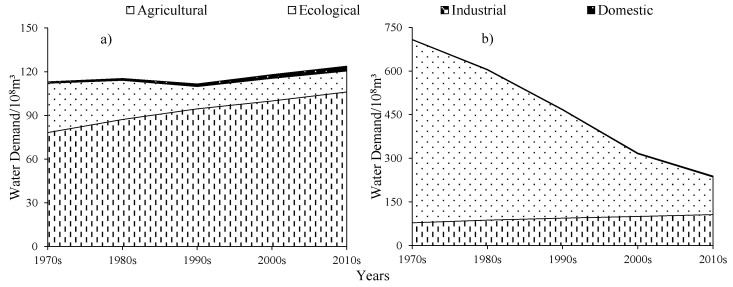
Total of water demand during the 1970s to 2010s. Notes: (**a**) Without EWD of Aral Sea; (**b**) With EWD of Aral Sea.

The Amu Darya River flows into the delta and eventually into the Aral Sea. As shown in Figure 9, the TWD with and without the EWD of Aral Sea has been calculated respectively, where only the EWD of the big Aral Sea was calculated after the split of the Aral Sea in 1987. Within the delta, the TWD has continued to increase due to the continued increase in AWD over the past 50 years, but the TWD continues to decrease within the LADB because the EWD of the Aral Sea has been decreasing sharply and accounts for the largest share of the TWD. Within the delta, the AWD was the main component of TWD and the average AWD was 93.17 × 10^8^ m^3^, which is continuously increasing at a rate of 0.68 × 10^8^ m^3^/y, and the EWD shows a continuous decreasing trend with a rate of 0.29 × 10^8^ m^3^/y. However, within the LADB, the EWD was huge with an average of 372.66 × 10^8^ m^3^, but decreases as the area and volume of the big Aral Sea gradually reduce with years with a rate of 15.51 × 10^8^ m^3^/y. IWD and DWD were very small, the sum of the two was about 2.02 × 10^8^ m^3^ and generally remains the same. 

### 3.3. Analysis of Balances of Supply-Demand

#### 3.3.1. Structural Composition

**Figure 10:**
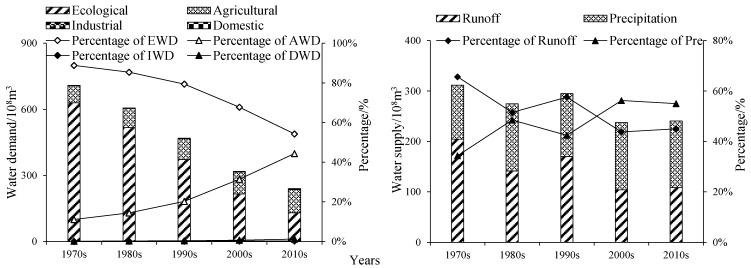
Composition changes of water demand and water supply in the LADB during the 1970s to the 2010s. Notes: EWD, ecological water demand; AWD, agricultural water demand; IWD, industrial water demand; DWD, domestic water demand.

The percentages of both supply and demand for water had changed significantly over the past 50 years. As shown in Figure 10. In the TWD, the percentages of EWD, AWD, IWD, and DWD were 88.82%, 11.04%, 0.02%, and 0.12% in the 1970s, respectively; and were 54.26%, 44.34%, 0.21%, and 1.20% in the 2010s, respectively. The percentage of EWD accounted for the largest proportion and decreased to 54.26% in the 2010s, while the percentage of AWD continued to increase, from 11.04% in the 1970s to 44.34% in the 2010s, and the percentages of IWD and DWD accounted for less than 2%. In the TWS, the main source of water supply experienced the transition from runoff to precipitation in the 2000s. Before the 2000s, the runoff was the main source of water supply, and the average percentage of runoff accounted for 58%; after the 2000s, the percentage of runoff has been fluctuated and decreased, and the precipitation became the main source of water supply, the average percentage of precipitation accounting for 56%.

#### 3.3.2. Gap of Water Supply and Water Demand

**Figure 11:**
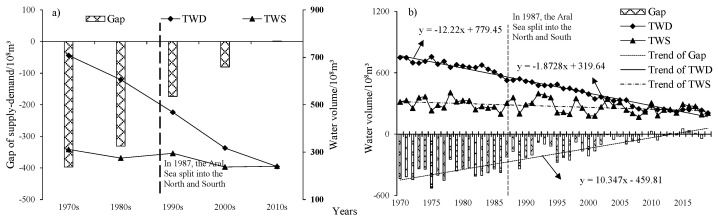
Variations of water supply-demand gap in the study area during 1970 to 2019. Notes: (**a**) Gaps of decade; (**b**) Gaps of years.

Analysis of the balance of water resources supply-demand, if only consider the scale within the UISs, there must be surplus. Therefore, the changes of water surplus and deficit in the South Aral Sea and the UISs as a whole were analyzed, results as shown in Figure 11.

In the past 50 years, the supply and demand of water resources of the LADB were generally in an unbalanced state, and water demand exceeded water supply except in the 2010s. The average water supply and water demand were 271.88 × 10^8^ m^3^, 467.85 × 10^8^ m^3^, respectively, both TWS and TWD had a significant trend of reduction with a significance of 99% by Mann-Kendall test, and the rate of reduction was 1.87 × 10^8^ m^3^/y and 12.22 × 10^8^ m^3^/y; the gap has a significant increasing trend with a significance of 99% and a growth rate of 10.35 × 10^8^ m^3^/y by Mann-Kendall test, and the average gap was −195.96 × 10^8^ m^3^, which means the state was deficit constantly in the study area. In addition, the gap of supply-demand gradually decreases with the time, which were −396.93 × 10^8^ m^3^, −330.70 × 10^8^ m^3^, −173.18 × 10^8^ m^3^, −80.48 × 10^8^ m^3^, and 1.47 × 10^8^ m^3^ in the 1970s, 1980s, 1990s, 2000s, and 2010s, respectively. In the annual analysis of water surplus and deficit, water shortages have existed in 43 of the past 50 years, with a historical guaranteed rate for water resources (the historical guarantee rate is the number of years in which the water demand can be satisfied as a percentage of the total number of years calculated) of 14%. The years of water deficits were 1970–2008, 2011–2012, 2014, and 2018. Among them, the proportion of water shortage years before 2010 accounted for 97.5%, and that after 2010 accounted for 40% (Table 5).

#### 3.3.3. Water Scarcity Index

**Figure 12:**
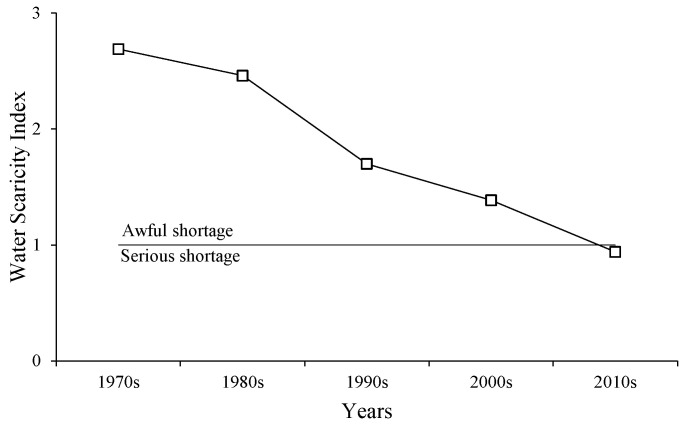
Distribution of WSI during the 1970s to 2010s. Notes: WSI, water scarcity index, over 1 means awful shortage of water resources in regional, and over 0.4 means serious shortage of water resources in regional.

The water demand was far greater than the available water resources in the LADB, especially in the 1970s and the 1980s. Although the WSI values in the region vary considerably among the decades, they were basically in a state of awful water deficiency (Figure 12). The values of WSI in the past five decades were 2.69, 2.46, 1.70, 1.39, and 0.94, respectively. The WSI value exceeds 1 were awful shortage and the value between 0.4 and 1 were serious shortage. Therefore, the water resources status of the region was awful shortage from the 1970s to the 2000s and was a serious shortage in the 2010s. The WSI analysis shows that the region has been experiencing water shortages for the past 50 years, awful shortages until the 2010s, mitigating to serious shortages after the 2010s. Based on the water supply and demand results in Section 3.3.2, although the TWS and TWD have reached a balance status in several years after 2010, the water surplus was only 8.61 × 10^8^ m^3^ to 52.11 × 10^8^ m^3^. Combined with the standards of WSI (Table 3), the current regional water resources are in a serious shortage, if the water inflow from the Amu Darya River decreases, or the water demand for ecological restoration continues to increase, the water resources in the study area will be in deficit again, so this paper considers that the current state of slightly surplus of regional water resources is extremely unstable. Therefore, this paper considers that the current regional water resources situation belongs to an extremely vulnerable state of a slight surplus.

## 4. Discussion

### 4.1. Uncertainties in the Calculation of Agricultural and Ecological Water Demand 

Agriculture and ecology were the main water users in the LADB, consuming more than 98% of water resources. Therefore, calculating agricultural and ecological water demand accurately determines the rationality of water supply and demand balance analysis. The Penman–Monteith method was used to calculate the crop water requirements in this study, considering the crop coefficients for each crop in different growth periods. The estimated ${ET}_{a}$ values for cotton, wheat, rice, vegetables, and melon were compared with the results in previous studies [9,10,11]. As shown in Table 6, the results showed reasonable ranges, although our estimated ${ET}_{a}$ values for melon were lower than those reported by them. Such differences may cause by multiple factors, such as changes in *K_C_* values, climatic conditions in different study periods, and uncertainties in the data input process, etc.

For the ecology sector, the EWD mainly includes water demand of natural vegetation, such as woodland, grassland, and wetland, and evaporation water demand of the Aral Sea. In this study, the water demand of natural vegetation was calculated by using the quota area method, which is widely used in arid areas with few observation data. The values of ecological water demand quota for natural vegetation refer to the research results in Tarim River Basin, which has similar conditions to the LADB [25]. The estimated evaporation of the Aral Sea was between 900 mm and 1300 mm, and Liu et al. [9], Benduhn and Renard [15], and Bortnik [43] estimated the evaporation of water bodies were between 1100 mm and 1500 mm, about 1100 mm, and 950–1050 mm, respectively, what’s more, Benduhn and Renard [15] estimated that the annual evaporation from the Aral Sea was 50.92 km^3^ during 1981 to 1990 years, the estimation result of this paper was 47.32 km^3^ during the same period. In fact, in the Aral Sea, the salinity of water also has a great impact on evaporation; however, due to the lack of experimental data, the influence of lake salinity is not considered in the current calculation.

### 4.2. Potential Impact of Environmental Protection Policy on Water Supply-Demand Balance in the Aral Sea Region

At present, water-saving irrigation technologies have been used the account for about 7% of the total irrigated area in Uzbek in 2020, with the introduction of water-saving technologies on 284 thousand hectares of land, where 114.2 thousand hectares are under drip irrigation technology. Khaydar et al. [10] indicated that the irrigation water use efficiency was about 35% in the irrigation in the NUKUS region. There is a huge waste of water in the process of agricultural production, especially in the irrigation areas in the LADB, and water scarcity needs to be alleviated by improving irrigation efficiency in the NUKUS region of Uzbekistan [10,44]. To improve the path of sustainable development of the country, the Republic of Uzbekistan has issued a series of organizational measures to fundamentally improve the country’s agricultural and water management systems. A decision was approved on the “Approval of the Strategy for the development of water resources management and irrigation sector for 2021–2030” in February 2021, which begins with increasing the share of concrete channels in the irrigation system from 35% to 38%, and it was planned to increase the total irrigated area with water-saving technologies to 2 million hectares by 2030, of which 600 thousand hectares will be under drip irrigation. On the other hand, the United Nations Development Program (UNDP) and Uzbekistan launched the Green Aral Sea Project in 2020, which can improve the lives of people in local communities by planting a 100-hectare forest of 100,000 saxaul saplings on the dry seabed. The plants will stop the movement of toxic salts and sands, helping reduce high rates of tuberculosis and other illnesses. 

Under such a policy background, the supply-demand relationship of water resources in the lower reaches of the Amu Darya River will face new challenges. The inflow into the LADB may increase when water-saving irrigation technology has been implemented widely in the irrigation area of the Amu Darya River Basin. The water demand of AWD may decrease with the water-saving policies, but it may also increase with the expansion of cultivated land. And the EWD of the Aral Sea region will increase with the promotion of the Green Aral Sea Project. On the premise of ensuring regional economic development and improving people’s lives, it is necessary to restore the ecological environment of the Aral Sea as much as possible. Optimal patterns of water resources allocation and crop construction should be the key content of water resources management in the lower reaches of the Amu Darya River in the future.

## 5. Conclusions

In this study, water demand and supply in the LADB were calculated over the past 50 years, and the variation of water resources consumption in multi-users and water supply from precipitation and streamflow were analyzed, and the balance and gaps between the water demand and supply were investigated. The findings revealed that both water supply and demand are decreasing with rates of −1.87 × 10^8^ m^3^/y and −15.59 × 10^8^ m^3^/y, respectively. The decrease in water demand was due to the decrease in EWD as the Aral Sea shrank, and the decrease in water supply because of the inflow from the Amu Darya River. The decrease of river inflow, coupled with the continuous increase of irrigation water, has exacerbated the process of shrinkage of the Aral Sea; the ecological water demand decreased significantly. The composition of the water supply and demand has changed, with the main water user shifting from the ecological to the agricultural sector, and the main water source shifting from runoff to precipitation. In recent 50 years, the average water shortage was 195.96 × 10^8^ m^3^, and the overall supply and demand for water resources was an unbalanced state in the study area, with water demand always exceeding water supply except for the 2010s. The WSI decreased from 2.69 to 0.94, and changed from awful to serious, which indicates that the water resources of the region have reached an extremely vulnerable state of a slight surplus.

Water resources are the primary limiting factor for the socio-economic development of the study area, and the fragile ecological environment of the arid zone is also the key to restricting economic and social development. Water resources are the focus of conflict between the development of both the economic and social systems and the ecological and environmental systems, and are also the link and bridge to coordinate the two systems. However, the allocation and security of water resources are exposed to risks arising from uncertainties in natural phenomena, social phenomena, and human activities, such as changes in precipitation runoff, population changes and economic development, policy changes, wars, and the limitations of human understanding of the objective world. Therefore, to maximize the economic benefits of regional water use under the uncertainty of water supply and demand, we should plan the water use of each sector rationally, and make scientific predictions and reasonable allocations of water resources in the region.

## Figures and Tables

**Table 1:** The detailed description of the data.

Data Types		Period	Data Sources/Description
LULC		1970, 1990, 2000, 2010, 2015	“Earth System Science Data Sharing Platform—Xinjiang and Central Asia Scientific Data Sharing Platform”
Crop planting pattern		1970–1987	Statistical Yearbook of the National Economy published year by year in the USSR from 1960–1987 provided by the Literature Information Center, Xinjiang Branch of the Chinese Academy of Sciences (http://3w.xjlas.ac.cn/category_59/index.aspx) (accessed on 19 August 2021)
		1988–1990	The Database Official Statistics of the Countries of the Commonwealth of Independent States. Annual statistics published in CD format
		1991–1999	The National Bureau of Statistics of the Republic of Uzbekistan (https://stat.uz/uz) (accessed on 19 August 2021)
		2000–2019	CAWA GREEN PROJECT (https://wuemoca.geo.uni-halle.de/app/#) (accessed on 19 August 2021)
Meteorological data		1970–2019	The National Oceanic and Atmospheric Administration (https://gis.ncdc.noaa.gov/maps/ncei/cdo/daily) (accessed on 19 August 2021) (http://data.ceda.ac.uk/badc/cru/data/cru_ts/cru_ts_4.04/data/) (accessed on 19 August 2021)
Water Resources Data	Domestic	1970–2019	The National Bureau of Statistics of the Republic of Uzbekistan (https://stat.uz/uz) (accessed on 19 August 2021) Ministry of Water Resources of the Republic of Uzbekistan (https://water.gov.uz/en) (accessed on 19 August 2021) ICWC (http://www.icwc-aral.uz/icwc_bulletins.htm) (accessed on 19 August 2021)
	Industrial	1970–1985	Based on linear trend extrapolation of 1986–2019 data
		1986–2019	the National Bureau of Statistics of the Republic of Uzbekistan (https://stat.uz/uz) (accessed on 19 August 2021) Ministry of Water Resources of the Republic of Uzbekistan (https://water.gov.uz/en) (accessed on 19 August 2021) ICWC (http://www.icwc-aral.uz/icwc_bulletins.htm) (accessed on 19 August 2021)
	Runoff	1970–2019	(http://www.cawater-info.net/water_quality_in_ca/syr_e.htm) (accessed on 19 August 2021)
	Area of Aral Sea	1970–2019	Benduhn and Renard [15]; Small et al., [16]; Crétaux et al., [17]; Zan et al., [18]

**Table 2:** Crop seasonal information in the study area.

Crop.	Vegetation Period			Kc			
	Planting Date	Harvesting Date	Days (d)	Initial Stage	Development Stage	Mid Season Stage	Late-Season Stage
Cotton	16 Apr	28 Oct	196	0.50	0.83	1.15	0.60
Wheat	15 Oct	10 Jun	238	0.40	0.78	1.15	0.25
Rice	11 May	6 Nov	179	1.05	1.13	1.20	0.70
Maize	12 Apr	8 Aug	118	0.30	0.75	1.20	0.60
Sunflower	11 Apr	25 Jul	105	0.40	0.78	1.15	0.55
Vegetables	21 Apr	11 Aug	112	0.70	0.88	1.05	0.95
Orchard	16 Mar	25 Sep	193	0.60	0.85	1.10	0.70
Melon	29 Apr	25 Aug	119	0.50	0.68	0.85	0.60

**Table 3:** Classification standard of WSI.

Range of *WSI*	Level of Water Shortage
0 < *WSI* ≤ 0.1	Surplus
0.1 < *WSI* ≤ 0.2	Slight shortage
0.2 < *WSI* ≤ 0.4	Moderate shortage
0.4 < *WSI* ≤ 1	Serious shortage
1 < *WSI*	Awful shortage

Note: The value of *WSI* must be greater than zero. *WSI*, water scarcity index.

**Table 4:** Statistical areas and percentages of different land use types.

Land Use Types	1970		1990		2000		2010		2015	
	km^2^	%	km^2^	%	km^2^	%	km^2^	%	km^2^	%
Cultivate	10,296.93	10.79	13,080.12	13.70	12,650.84	13.25	13,199.78	13.83	13,908.31	14.57
Woodland	3452.36	3.62	1112.80	1.17	979.90	1.03	1009.70	1.06	983.36	1.03
Grassland	12,782.60	13.39	12,899.22	13.51	13,994.22	14.66	12,913.90	13.53	12,018.62	12.59
Urban land	699.24	0.73	611.46	0.64	1303.33	1.36	1419.04	1.49	1612.79	1.69
Water	61,717.52	64.65	35,288.20	36.97	22,089.12	23.14	9480.07	9.93	5325.77	5.58
Wetland	1698.97	1.78	2193.14	2.30	3394.82	3.56	2674.82	2.80	3555.08	3.72
Unutilized land	4810.72	5.04	30,273.39	31.71	41,046.09	43.00	54,761.01	57.37	58,054.38	60.82

**Table 5:** Water shortage and guarantee rate in years.

Years	Counts of Year of Water Shortage	Water Shortage Volume /10^8^ m^3^	Historical Guarantee Rate/%
1070s (1970–1979)	10	396.93	0
1980s (1980–1989)	10	330.70	0
1990s (1990–1999)	10	173.18	0
2000s (2000–2009)	9	89.45	10
2010s (2010–2019)	4	29.72	60

**Table 6:** Comparison of the average crop water requirement (CWR) values for main crops in the study area.

References	Periods	CWR/mm				
		Cotton	Wheat	Rice	Vegetable	Melon
Schieder [42]	2003	762.2	509.2	758.0	866.9	760.4
Bobojonov [11]	2006–2007	799.0	383.0	1050.0	619.0	625.0
Liu [9]	2018	866.5	438.3	950.0		
Khaydar [10]	2004–2017	887.2	492.0	1002.1	619.1	640.2
This study	1970–2019	792.89	553.8	941.21	646.09	536.2

## Data Availability

All data used in this study are openly available from sources quoted in the text.
